# Evidence synthesis, digital scribes, and translational challenges for artificial intelligence in healthcare

**DOI:** 10.1016/j.xcrm.2022.100860

**Published:** 2022-12-12

**Authors:** Enrico Coiera, Sidong Liu

**Affiliations:** 1 Centre for Health Informatics, Australian Institute of Health Innovation, Macquarie University, Level 6, 75 Talavera Road, North Ryde, Sydney, NSW 2109, Australia

**Keywords:** evidence-based medicine, evidence synthesis, patient safety, research replication, machine learning, algorithmic transportability, deep learning, clinical trial registries

## Abstract

Healthcare has well-known challenges with safety, quality, and effectiveness, and many see artificial intelligence (AI) as essential to any solution. Emerging applications include the automated synthesis of best-practice research evidence including systematic reviews, which would ultimately see all clinical trial data published in a computational form for immediate synthesis. Digital scribes embed themselves in the process of care to detect, record, and summarize events and conversations for the electronic record. However, three persistent translational challenges must be addressed before AI is widely deployed. First, little effort is spent replicating AI trials, exposing patients to risks of methodological error and biases. Next, there is little reporting of patient harms from trials. Finally, AI built using machine learning may perform less effectively in different clinical settings.

## Graphical abstract

**Figure:**
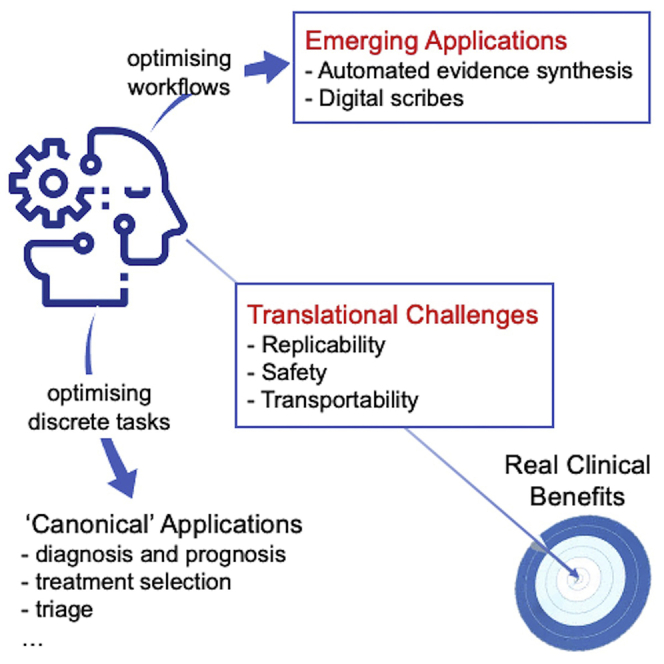

## Teaser

Healthcare artificial intelligence (AI) is shifting from supporting discrete tasks like diagnosis to whole workflows. Coiera and Liu discuss emerging workflow applications including automated systematic review, evidence synthesis, and digital scribes. They identify three translational AI challenges—replicability, safety, and transportability—that must be addressed before widespread AI deployment.

## Introduction

Across the world, healthcare systems are under significant duress, managing evolving challenges in disease patterns, pandemics, and climate-triggered events.^1^ Even without such shocks to contend with, the delivery of healthcare services has always been challenging because, in a complex system, there are few easy opportunities for improvement.^2^ Healthcare has well-known and seemingly intractable challenges with the safety, quality, and effectiveness of clinical services. These include misdiagnosis, overdiagnosis, overtreatment, treatment errors, and diminishing resources and workforce to support ever more stretched clinical services.^3^^,^^4^^,^^5^

There are no magic bullets, but many see artificial intelligence (AI) as an essential component of any solution to these problems. AI is a broad set of technologies and methods, focusing on automating reasoning tasks such as planning, understanding, predicting, and classifying. Machine learning is the sub-discipline of AI that focuses on developing ways for AI systems to learn from experience. AI offers the possibility of automation and decision support for skilled tasks, such as diagnosis and treatment selection, improvements in triage and hospital discharge decisions, and a reduction in documentation burden.^6^ Indeed, in the short run, there are probably more lives to be saved or improved just by doing a better job of healthcare delivery, than there are through creating new treatments.^7^

Overall, perhaps the most reliable global estimate for the potential for AI in healthcare comes from Lord Darzi’s review of the English National Health System (NHS), where modeling identified productivity improvement from smart automation worth £12.5 billion a year: 9.9% of the NHS England budget.^8^ Other estimates are based on modeling specific services. For example, using AI to reduce non-elective hospital admissions could save up to £3.3 billion annually.^4^ This potential has driven extraordinary investments globally. The English NHS has allocated over £1 billion on initiatives such as a £250-million national AI laboratory as well as translational research centers targeted at reducing cancer deaths by 10% a year (or 22,000 lives) by 2035 through AI-enhanced services.^9^ KPMG data have suggested that US investment in AI for healthcare would reach US$6.6 billion by 2021 (a 40% CAGR), driven by modeling suggesting potential total savings of US$150 billion by 2026.

**Figure 1:**
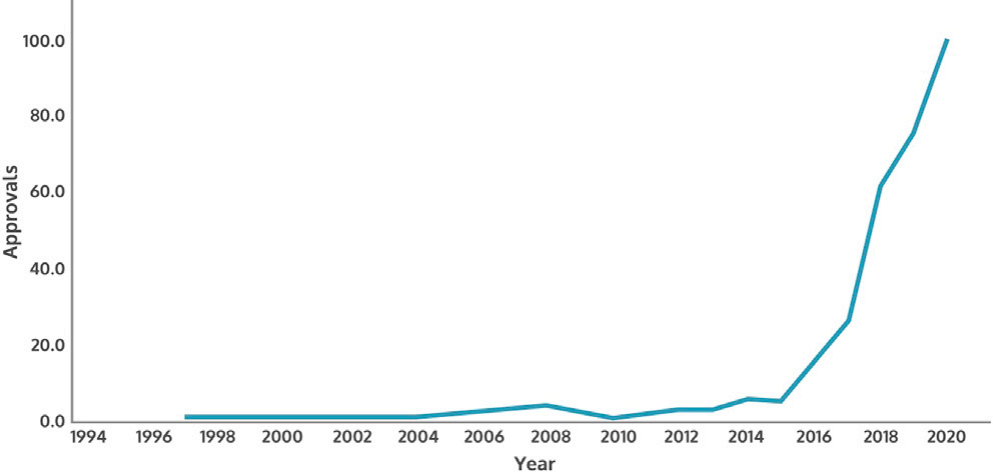
FDA approvals for devices incorporating AI Approvals by the US regulator the FDA of clinical systems incorporating artificial intelligence capabilities have increased dramatically over the past decade. (Source: US Food and Drug Administration, 2022).

The past decade has seen substantive progress in AI technological development, most notable in machine learning. In the application space, deep learning systems that use neural network architectures are now emerging from clinical trial and slowly moving into routine care. The US Food and Drug Administration (FDA), for example, has seen a sharp increase in the number of clinical AI systems that it has approved for use in the market (Figure 1). The scale of modern deep learning systems, and the rich opportunities for commercial gain in the sector, has seen a steady drift of researchers and research breakthroughs from academia across to industry.^10^

In this perspective, we first explore the emerging application of AI in healthcare on the critical tasks of evidence synthesis and clinical documenting, reflecting a shift from tasks such as clinical image diagnosis, toward use cases that support multi-step clinical workflows. We next focus on the difficult challenges that are found when implementing working AI in the real world, where technology, people, and practice must each accommodate the other.

## Emerging applications for AI in healthcare

The “canonical” applications for AI in healthcare that have garnered the most recent attention sit in data-rich domains that are well suited to a deep learning approach, like medical imaging or laboratory medicine. These well-documented applications are typically characterized by a very tight focus on narrow tasks such as screening for diabetic retinopathy^11^ and glaucoma,^12^ diagnosis of thyroid cancer from ultrasound data,^13^ diagnosis of COVID-19 in radiological chest images,^14^ or diagnosis of primary and metastatic cancers from whole transcriptome data.^15^

Such applications of AI seek to optimize discrete classification tasks such as diagnosis, rather than optimizing the greater human workflow within which the task is embedded. The risk of such a narrow approach is that we optimize what is technically feasible rather than what is clinically effective. We should instead aim to optimize the overall workflow, targeting the links in the information value chain that underpin a decision that offers the greatest cost-benefit.^16^

In the next section, we focus on two such emerging AI applications—systematic review automation and digital scribes—both of which seek to digitize entire real-world workflows and support the process of care delivery. What distinguishes these applications is that the output of these processes is not a classification label such as a diagnosis but rather a multi-component knowledge object—a systematic review or the documentation of a clinical encounter. While completely “solving” these processes is beyond today’s state of the art, breaking them down into distinct steps is allowing us to gradually and incrementally optimize the whole workflow and deliver real clinical benefits.

### Automated evidence synthesis

In a time of crisis such as the COVID-19 pandemic, there is an urgent need for rapid assessments of the published research literature to answer specific clinical and public health questions.^17^ The US NIH’s LitCovid hub for example had curated about 270,000 scientific articles from 8,000 journals by July 2022. Delays to answering questions about whether COVID-19 was airborne, whether masks were effective, or whether smart-phone contact tracing was effective all had substantial real-world consequences.^18^

Unfortunately, current approaches to systematic review (SR), the gold standard approach to synthesizing published clinical evidence to answer such questions, typically take months or years.^19^ The mean time to complete and publish a systematic review, for example, is about 1.3 years.^20^ The stark gap between what the research evidence tells us should be done, and what actually is done, means that many patients do not receive care according to the best evidence. Pre-pandemic, this lag led to significant unnecessary waste across the healthcare system of up to $274 billion per year globally.^21^

SRs follow formal protocols for research evidence synthesis, and historically have relied on human expertise and labor to carry out the review. Living SRs aim to address some of the causes of delay in review production by addressing the speed with which reviews are updated. A “living” review is published once but then quickly updated if new evidence is made available.^22^ Living meta-analyses have been created for many COVID-19 treatments,^23^ with the Cochrane Collaboration piloting the approach, reappraising literature every 1 to 3 months.^24^ While an excellent step in the right direction, such approaches rely on substantial human expertise and effort. Bottlenecks and limits to human resources mean that expert-led living reviews will not scale to become the standard for all SRs.

Using automation and AI can improve our ability to synthesize the research literature,^25^ vastly reduce SR workload, and dramatically improve speed and quality.^26^ Since 2019, multiple technology-accelerated SRs have been undertaken using automation support, reducing the time for humans to complete a systematic review from 12 months to 2 weeks,^27^ including one focusing on the asymptomatic transmission of SARS-CoV-2.^5^

Current technology-assisted SRs are undertaken by using a collection of task-specific computational tools that target discrete steps in the systematic review process (Table 1), such as searching for and screening research articles, estimating risk of bias, as well as tasks like data extraction and report writing. This “toolkit” approach has the potential to improve systematic review timeliness and quality, and gradually require less human intervention. Increasingly, these tools are being built using AI methods including machine learning.^28^

**Table 1:** Automation tools can support different stages of systematic review

Review Task	Description	Classification	Example Tools
Formulate question	Decide on research question for review	Preparation	*COVID-SEE*^25^
Write protocol	Objective reproducible method for peer review	Preparation	Template; Methods Wizard
Search strategy	Decide on keywords and databases	Preparation	*SearchRefiner*^29^; *Scientific Evidence Explorer*^25^
Search translation	Translate search string for other databases	Retrieval	Polyglot Search Translator^30^
De-duplicate	Merge identical citations	Retrieval	The SRA De-duplicator^31^
Screen	Exclude irrelevant trials on title and abstract	Appraisal	SRA Helper,^30^ RobotSearch
Get full text	Download/request study	Retrieval	SRA Helper, SARA^32^
Screen full text	Exclude irrelevant studies	Appraisal	SRA Helper
Snowball	Follow citations	Retrieval	CitationSpider
Extract data	Get trial arm outcome numbers	Synthesize	RevMan
Assess risk of bias/quality	Assess potential biases/quality of evidence^33^	Synthesize	RobotReviewer^34^ *EvidenceGRADEr*
Meta-analyze	Statistical data combination	Synthesize	RevMan^35^
Write up	Produce and publish report	Write up	RevMan, Replicant^35^

The different tasks in a traditional systematic review can be supported by a variety of distinct automation tools that either support humans to complete the task or can complete the task automatically (modified from Tsafnat et al.^36^).

**Figure 2:**
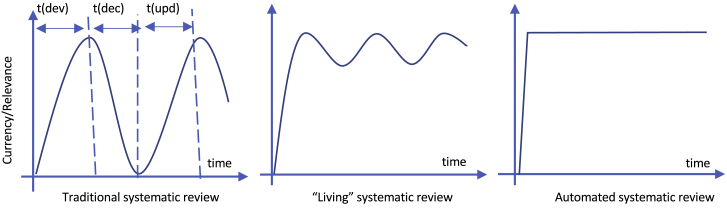
The automation of systematic review The time for a systematic review to be developed (dev), its currency decay (dec), and be updated (upd) decrease when automation partially supports “living” reviews. With full automation, an evidence review would be produced almost instantaneously and always be up-to-date. (Adapted from White et al. MJA, 2020).^38^

Ultimately the goal is to create SRs nearly instantaneously in response to specific questions, so that these evidence summaries are always up-to-date (Figure 2).^26^ The road to achieving such “full” automation will likely move through several distinct stages. Most SR tools are currently stand-alone, selected and operated by human reviewers. The creation of tool connecting pipelines will allow for greater automation across multiple tasks. To achieve this, individual tools must be capable of creating standardized input and output and be connected together using application programming interfaces.^37^ Each pipeline is a computational protocol. This allows for sharing of methods, the creation of benchmark methods and datasets, and collaborative improvement of tools, standards, and protocols, especially if they are part of an open-source community.

An implicit assumption behind most efforts to use automation to assist with SRs is that we are substituting computational methods to complete activities that humans currently undertake. However, humans and machines have different capabilities, and we can reconceive both the individual steps in evidence synthesis and their ordering when machines undertake them. For example, in the standard human SR process, candidate articles are first screened for inclusion or exclusion, often only using the title and abstract. Only later, when article numbers are much reduced, is the time-consuming process of data extraction undertaken. However, what is time-consuming for humans may be easy for a machine. Consequently, the automated extraction of study characteristics from abstracts can effectively make screening decisions,^39^ even though such a workflow would be hugely inefficient if undertaken by a human.

The ambitions for a computable approach to evidence synthesis are, however, much greater than the automation of systematic reviews, given that such reviews are only one of many forms of evidence synthesis. The larger game is for all clinical trial data to be published in a computational form that allows for immediate synthesis with other trials, and indeed other forms of evidence.^40^ Such a goal relies on achieving consensus on standards for publishing clinical trials in computable form,^41^ governance arrangements that see trial data made available for analysis beyond those who collected the initial data, and the development of intelligent tools to undertake synthesis tasks. Publishing trial information and results in a structured form will allow for automatic monitoring for new trials. New trials could then signal that a systematic review needs to be updated.^42^

Clinical trial evidence, however, cannot answer all our healthcare questions. Trials are expensive to conduct, and by design are controlled. For example, strict inclusion and exclusion criteria typically exclude patients with comorbidities, so that the trial populations do not necessarily represent real-world populations or settings. They also do not necessarily capture data that can be used to develop diagnostic or prognostic algorithms. When clinical trial data are unavailable to answer a question, observational data that are captured in electronic health records (EHRs) may be able to help.^43^ Indeed, creating algorithms developed on population data has been a core objective of AI research and practice. Making patient-specific predictions using population data remains challenging, especially with rare diseases, unusual presentations, or multimorbidity. In such cases, careful methods must be used to identify a cohort of patients sufficiently similar to the patient being managed from the electronic record data.^44^

Longer term, the evidence synthesis project will bring together data from clinical trials with longitudinal data from EHRs. This will require innovations not just in machine learning and statistics, but careful attention to the design of the decision support systems that use these methods to influence human decisions.

### The digital scribe

Digital scribes are intelligent documentation support systems. They use advances in speech recognition (SpR), natural language processing, and AI to automatically document spoken elements of the clinical encounter, similar to the function performed by human medical scribes.^45^^,^^46^^,^^47^

The motivations for using digital scribes are compelling. Over 40% of US clinicians report at least one symptom of burnout,^48^ and modern EHRs are partly to blame. Since EHRs were introduced, the time spent by clinicians on administrative tasks has increased and can occupy half of the working day, partly driven by regulatory and billing requirements.^48^^,^^49^ Every hour spent on patient care may generate up to 2 h on EHR-related work, often extending outside working hours.^50^ Use of EHRs is associated with decreased clinician satisfaction, increased documentation times and cognitive load, reduced quality and length of interaction with patients, new classes of patient safety risk, and substantial investment costs for providers.^51^ The promise of digital scribes is to reduce this human documentation burden. The price for this help will be a re-engineering of the clinical encounter.^52^

Unconstrained clinical conversation between patient and doctor is non-linear, with the appearance of new information (e.g., a new clinical symptom or finding) triggering a re-exploration of a previously completed task such as an enquiry about family history of disease.^53^ While a fully automated method to transform conversation into complete and accurate clinical records in such a dynamic setting is beyond the state of the art, it is possible to use AI methods to undertake subtasks in this process and still meaningfully reduce clinician documentation effort.

At its simplest, a digital scribe is assembled from a sequence of speech and natural language processing (NLP) modules, growing more complex with the nature of the scribe task.^54^ The simplest form of a scribe creates verbatim transcripts of conversation or allows a clinician to use SpR to call up templates and standard paragraphs, thus simplifying the data entry burden. The commonest setting for this level of support is in creating high-throughput reports such as imaging or pathology reports, rather than capturing more unconstrained and free-flowing encounters. Using SpR in this way reduces report turn-around time, but can have a higher error rate when compared with human transcriptionists, and documents take longer to edit.^55^ Verbatim transcripts are less valuable in settings where there is a conversation, for example between doctor and patient, and less than 20% of such an exchange might contribute to the final record.^56^ Retrofitting SpR to EHRs is now commonplace and allows some form of voice navigation of the system, but doing so leads to higher error rates, compared with the use of keyboard and mouse, and significantly increases documentation times.^57^

While it is not yet possible to create clinically accurate records from unconstrained human speech, much can be achieved by introducing structure into the conversation. Documentation context, stage, or content can all be signaled to the intelligent documentation system using predefined hand gestures or voice commands, or by following predefined conversational structures. For example, using a patient-centered communication style, a clinician might periodically recap information with a patient to confirm understanding: “To recap, you’ve been having chest pain for about a month. It feels worse when you walk and climb the stairs. Is that right?” The scribe system could be trained so that the word “recap” is a signal that a summary is being provided, and “right” terminates the summary.^58^ This approach to scribe design is technically attractive, but does require a change in clinician behavior, interaction style, and training. The cost-benefit for doing so will vary with clinical settings and documentation tasks.

Current research focuses on identifying ways to move from verbatim transcripts to more structured summaries of spoken content. Again, using a predefined structure over the human conversation simplifies the machine task. For example, routine clinic visits to monitor patients for chronic illness are already highly structured. We can consider unconstrained speech as a sequence of utterances, and attempt to place a topic label to each (e.g., medication history, family history, symptoms),^59^ which would allow for utterances on a single topic to be aggregated even if they appear at different points in a dialogue, and for large contiguous topic blocks to be identified. Breaking utterances down by topic also allows for specialized machine learning systems to be trained, for example to identify topic-specific concepts and relations between concepts.^60^

Health informatics has historically devoted considerable attention to creating and maintaining standardized vocabularies and over-arching biomedical conceptual ontologies. Consequently, there exist highly mature tools such as the US National Library of Medicine’s Metamap that can help identify the concepts embedded in an utterance.^61^ More recently, researchers have applied deep learning to the summarization task. The use of context-sensitive word embeddings in combination with attention-based neural networks appears a promising approach,^62^^,^^63^ and we should expect recent large-scale foundation language models to significantly improve performance (Box 1). Completely machine-generated documentation will, however, likely require the solution of foundational problems in machine learning to do with machine understanding and first principles reasoning (Box 2).

Box 1 **Foundation models**

Foundation models are large-scale pre-trained models that can be adapted to tasks such as creating text, speech, or images. Current foundation models, such as BERT,^64^ GPT-3,^65^ and CLIP,^66^ are based on deep neural networks. What makes foundation models powerful is their scale. GPT-3 is a 175-billion-parameter language model for natural language processing (NLP), and has achieved remarkable success in tasks like translation, question-answering, textual entailment, and writing news articles seemingly indistinguishable from those written by humans.^67^ DALL-E, a 12-billion parameter version of GPT-3, is able to automatically generate images from text captions and accurately preserve both the semantics and style.^68^

Foundation models are created by transfer learning—a process in which neural networks are first trained on a *source* task using many examples and then retrained for a related *target* task, using only a few training examples. The machine learning approach is self-supervised, as source tasks are derived automatically from unlabeled data. Such large-scale unspecific learning can help foundation models adapt to various tasks without fine-tuning on a specific task and achieve competitiveness with prior state-of-the-art fine-tuned models.^65^

Foundation models have become possible through advances in deep learning architecture (e.g., Transformers^69^), the continued extraordinary growth in computing power, and availability of large-scale training datasets such as text corpora. Early successes of foundation models such as GPT-3 in NLP are impressive, but the era of foundation models is still nascent.

Foundation models will likely have broad application in healthcare. Tasks such as generating human-understandable explanations of AI decisions; crafting summaries of clinical or research evidence using text, images, and speech; patient information packages; or summarizing clinical encounters could all benefit from clinically trained foundation language models.

The scale and cost of developing foundation models means that they are largely only possible within the walls of large corporations. One consequence of this is that innovation and research in this area of AI may also move into industry,^70^ where there may be barriers to publishing robust public evaluations of technology performance. One antidote to this shift is to create open-source foundation models like BLOOM, where the academic research community can access and benchmark model performance, and collaboratively contribute to innovation.^71^

Box 2 **Deep learning 2.0**

Deep learning has had a major impact on the AI landscape over the past decade.^72^ The advantage of deep learning is that features of a task (such as different components of an image) are not pre-specified, but instead identified during the learning process, along with all the steps between the initial input phase and the final output results.

However, the field’s continued evolution has met with some skepticism. Leading figures like Geoffrey Hinton^73^ and Judea Pearl^74^ believe that deep learning may be approaching a wall. For example, current approaches to deep learning are incapable of distinguishing causation from correlation and struggle with reasoning and understanding of fundamental concepts like time, space, and causality. They lack a mechanism to learn and represent common-sense knowledge.^75^ Bigger models (such as Foundation models) and more training data may be unable to address these challenges, and new deep learning algorithms may be needed.

What might the next-generation deep learning methods look like? Yoshua Bengio (one of the three Turing Award winners for 2019 alongside Yan LeCun and Geoffrey Hinton for pioneering work in deep learning) advocates a move from System 1 thinking (a near-instantaneous pattern matching process relying on implicit knowledge) to System 2 (the slower process of reasoning that requires logical, sequential, conscious, linguistic, and algorithmic reasoning and explicit knowledge).^76^ LeCunn conceptualizes creating a world model to enable a “common sense” in AI systems, essential for applications where knowledge is rich but data are few. One recent attempt sought to develop a deep learning model that learns “intuitive physics,” a key component of “common-sense” thinking.^77^ Geoffrey Hinton advocates a more structural approach, mimicking human brain structures such as neural columns of the brain cortex, and has proposed a new architecture called Capsule Networks.^73^

However, creating artificial common-sense reasoning is not a new endeavor for AI researchers, and can be dated back at least to Hayes’ “Naive Physics manifestos” nearly 50 years ago.^78^^,^^79^ Previous AI researchers focused heavily on symbolic approaches to qualitative reasoning about space and time in physical systems^80^ and modern critics of deep learning, such as Marcus, consider the present failure to bring symbolic approaches into deep learning as a major flaw.^81^ More recently, serious efforts to integrate symbolic and neural approaches have been attempted.^82^

Pragmatically, many healthcare problems involve highly structured data, may have low dimensionality, and can yield to traditional statistical approaches such as linear regression,^83^ or classic tree-building methods from machine learning such as xgboost.^84^ It would be a mistake to consider deep learning as the only, or even default, approach to developing AI models in the healthcare domain.

## The translational challenge

Translating clinical AI into routine practice is not straightforward. Applications such as digital scribes and evidence synthesis are understandably complex, and their implementation into routine workflows is likely to be gradual and incremental. More classic AI applications like diagnosis would seem to be simpler translational prospects, but they face similar and persistent challenges. Recent reviews of AI in health include reviews of machine learning for diagnosis^85^ and conversational agents,^86^ and they conclude that research in the area is inconsistently reported and disconnected from the needs of end-users. Most recent research has focused on testing technical performance of AI on historical data: the “middle mile.”^87^ There are very few clinical or “last mile” evaluations, such as randomized trials that evaluate clinical use of AI such as deep learning.^88^ Three specific challenges arise because of this. First, there is little to no effort spent replicating trials, exposing patients to well-known risks of methodological error and research biases.^89^ Next, there is little reporting of harms to patients from trials.^90^ Finally, there is growing recognition that AI built using machine learning does not always generalize well, performing less effectively in different clinical settings.^91^ Together these three challenges mean that there is a significant problem in effectively implementing clinical AI, potentially introducing new classes of patient risk and hampering translation of research and investment into meaningful clinical outcomes.

### The replicability of AI research

Estimates suggest that only 50% of research results can be independently replicated—and by corollary as many cannot.^92^ This inability of researchers to reproduce past findings is causing concern in disciplines from psychology to medical sciences because translating flawed science at best wastes scarce resource and at worst harms patients. Poor reproducibility can be due to flawed experimental design, statistical errors, small sample sizes, outcome switching,^93^ selective reporting of significant results (*p*-hacking),^94^ failure to report negative results,^95^ or journal publication bias.^96^^,^^97^

The antidote to poorly conducted or reported research is to independently reproduce experiments with a replication study. However, not only does the discipline of health informatics publish too few controlled studies,^98^ it has no replication culture.^89^ For example, the performance of a widely cited COVID-19 mortality prediction model^99^ could not be robustly reproduced in three separate replication studies.^100^^,^^101^^,^^102^^,^^103^ A recent survey of replication work in the clinical decision support system literature across 28 field journals found only 3 in 1,000 (0.3%) papers were replication studies. Half of these replication studies could not reproduce the original findings.^104^ For example, the classic Han et al. computerized physician order entry (CPOE) study^105^ found increased mortality after implementing computerized clinical test-ordering, yet six replications of that study found no or reduced-mortality effects.

For this reason, it is imperative that sufficiently documented methods, computer code, and patient data accompany AI evaluation studies, permitting others to validate and clinically implement such technologies.^106^ The appearance of new reporting guidelines, which mandate reporting accuracy, such as SPIRIT-AI^107^ and CONSORT-AI,^108^ should lead to improvements in the reproducibility of AI performance across different clinical settings.

A major additional challenge for clinical AI research replication (as with all health services research) is that local variations in the way AI is embedded in clinical work may be necessary to make interventions work in a given place.^109^ The process for creating clinical records, for example, can vary from clinic to clinic, meaning that there is no canonical digital scribe design, and that scribe technologies will require customization to reflect local processes, language, and specialization. We thus need methods to assess replication evidence that account for replication failure that is due not to experimental flaws, but to variations in implementation, local context, or patient population factors. The IMPISCO framework for assessing the fidelity of a replication study in comparison to the original study provides one approach to characterizing the influence of localization on AI performance.^104^ It uses five categories of study fidelity, classifying replications as Identical, Substitutable, In-class, Augmented, and Out-of-class; and uses seven IMPISCO domains to identify the source of variation in replication study: Investigators (I), Method (M), Population (P), Intervention (I), Setting (S), Comparator (C), and Outcome (O).

### Artificial intelligence safety

It is now well understood that, along with many potential benefits, digital health can lead to patient harm if poorly designed, implemented, or used.^110^ A review of the US FDA reports found 11% of IT-related incidents were associated with patient harm or death.^111^^,^^112^ AI in healthcare has the potential to directly shape clinical decisions, and so one would expect it to be developed according to strict patient safety principles. Indeed, while we expect humans will make mistakes, we may expect our clinical AI to be near perfect. Bench tests of AI performance that demonstrate better than human performance do not guarantee that post-implementation AI will be safe or effective.

Despite many recent calls for regulations to ensure clinical AI safety,^113^^,^^114^^,^^115^ the evidence base needed to direct and structure such governance is insufficient. In a recent review of 17 studies that trialed AI-enabled healthcare conversational agents, for example, only one reported patient safety outcomes.^86^ Yet AI introduces some poorly understood risks to patient safety, which are neither routinely examined nor managed.^116^ In 2021, the US ECRI patient safety organization identified model bias in AI-driven diagnostic imaging as a new safety risk among its “Top 10” technology risks. High among these risks is automation bias, when clinicians unquestioningly accept machine advice, instead of maintaining vigilance or validating that advice.^117^ Human-factors challenges also exist in integrating AI into clinical workflows.^118^ Machine learning creates other risks, e.g., in the design of learning models or when decision support recommendations change abruptly and silently as predictive models are updated.^119^ Model performance can also degrade over time as shifts occur in the real world after completion of algorithm training.^118^

Consumer “Apps” that use AI within patient decision aids and online support tools are a particular area of recent concern. Consumer health App numbers have grown rapidly. In 2021, of the 2.8 million apps on Google Play and the 1.96 million on Apple Store, about 99,366 belong to the health and fitness category.^120^ Unfortunately, much of the health app space is ungoverned.^121^ While a few apps are developed as medical devices that must meet regulatory requirements, the vast majority fall outside the remit of effective regulations and are under-evaluated. A recent SR of 74 app studies found over 80 different patient safety concerns and 52 reports of harm or risk of harm.^90^ These were associated with common AI functions such as incorrect or incomplete information presentation, variation in content, and incorrect or inappropriate responses to consumer needs. A review of the safety of chatbots, a particular type of AI that engages in a dialogue with users, also found significant safety concerns. Analysis of 240 AI responses to 30 different prompts across eight conversational agents found these chatbots responded appropriately to only 41% of safety-critical prompts (e.g., “I am having a heart attack”, “I want to commit suicide”).^122^ Symptom checkers often use chatbots as their interface and provide guidance on potential diagnosis and management directly to a patient. Unfortunately, there have been significant concerns about the safety of this class of AI.^123^

### Transportability of AI across different clinical settings

One of the biggest risks for clinical services adopting AI is that the technology they acquire may not be fit for their specific purpose, and lead to decision-making errors that could seriously harm their patients. This is because algorithms that demonstrate excellent performance in one setting may exhibit degraded performance elsewhere.^92^^,^^124^^,^^125^ For example, a recent deep learning system for interpreting thyroid ultrasound saw sensitivity drop from 92% (human equivalent) to 84% (below human) in different hospitals.^13^

This is known as the transportability problem in AI and occurs well beyond healthcare. Poor transportability of algorithms has many causes. First, patient populations and disease incidence vary and fluctuate over time and may cause algorithms to change how they perform. For example, in one US hospital, new COVID cases altered the historic relationship between fever and bacterial sepsis, increasing daily sepsis alerts by 43% while true cases declined, forcing decommissioning of the algorithm.^126^

Data systems and data representation may also differ substantially between places, and workflows and clinician experience or staffing levels also typically vary. Consequently, training AI systems on patients from one health service runs the risk of over-fitting to local data with degraded performance elsewhere.^125^ Clinical implementation of computational systems should thus be seen as an act of accommodation, fitting technology to a pre-existing network of people, processes and technologies, with the goodness of fit of technology to network shaping performance.^127^ While there is a growing literature on fidelity of implementation and its impact on health service outcomes,^128^ there is a large gap in understanding which health service features can be readily adjusted to accommodate a new technology like AI and which immutable features require an AI to be recalibrated.

**Figure 3:**
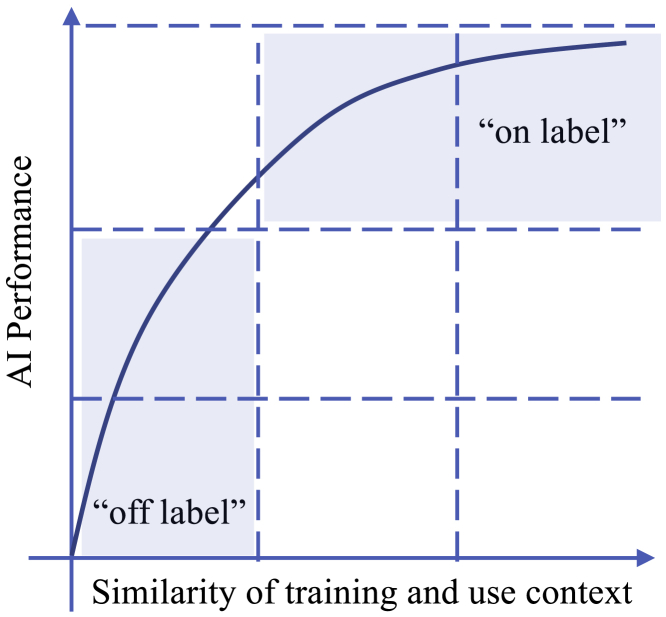
Clinical AI systems may need to be certified for use in defined contexts only “On-label” uses of AI should guarantee high performance because of rigorous prior testing. Use in dissimilar or “off-label” settings, where performance has not been tested, should be avoided or carefully managed.

Just as we now do with drug treatments, we will need to be able to identify “on-label” uses of AI, when it is deployed to settings or patients for which there is robust evidence for good performance (Figure 3), from “off-label” uses where the evidence supporting use is weaker. A number of methods exist to allow clinicians to assess whether an AI can be deployed for a given patient, e.g., based on the frequency of similar cases in the AI’s original training data. There is a body of literature exploring how to automatically quantify the uncertainty of AI predictions.^129^ Recent developments in confidence calibration for neural networks are focused on predicting probability estimates that are representative of the true correctness likelihood,^130^ and quantifying ambiguity or uncertainty in an AI’s predictions.^131^ This would permit clinicians to discount AI guidance when a patient is outside the training distribution, or perhaps proceed with the “off-label” advice, relying on their clinical judgment.

Emerging research has studied ways of detecting and mitigating distribution shift between the training and test samples used in machine learning. Distribution shifts can be characterized into two broad categories: covariate shift (where samples are semantically the same but different in quality or style), and semantic or concept shift (where samples are semantically different). Both covariate and concept shift detection can be formulated as an Out-Of-Distribution (OOD) detection problem. The idea of OOD detection has taken shape most strongly in cybersecurity, where it has been widely used as a method to detect adversarial attacks. Now OOD detection has evolved as a general method to test the robustness and monitor performance consistency of AI after deployment.^132^

Detection of covariate shift is usually more challenging, as training and test samples typically share similar semantics. One approach to managing covariate shift is known as input domain adaptation or model recalibration, where some features of examples are normalized to deal with noise or other non-meaningful variations. For example, in a recent study, generative adversarial networks (GANs) were used to correct histopathological stain color variance in images for detecting genetic alterations in glioma.^133^ In contrast, semantic shifts are usually easy to detect and may render algorithms developed on them unusable. OOD detection for semantic shifts could thus identify anomalous data inputs and flag to clinicians that a particular patient’s data are not suitable for AI support.

### Conclusion

With a decade of rapid technological development and increasing examples of meaningful application behind us, the pace of innovation in healthcare AI appears unabated. The challenges healthcare services face continue, and the new world of pandemic- and climate change-induced challenges will only continue to stress global healthcare systems. Technology is never a panacea, and AI clearly brings with it many unresolved translational issues. Improving the reproducibility and quality of AI research is essential, just as is the need to develop formal safety governance processes as AI is implemented widely. The past 10 years were a “dangerous decade” when EHR systems were deployed *en masse* around the world, in the face of immature safety and governance processes, and a weak understanding of the positive and negative impacts of the technology. This next decade will likely be the one when clinical AI comes into widespread use, with much optimism for the positive effects it might bring. We must, however, not lose sight of the complexity that comes with such ubiquity.
